# Editorial: Nephrotoxicity of immune checkpoint inhibitors

**DOI:** 10.3389/fmed.2023.1347101

**Published:** 2024-01-09

**Authors:** Tess Van Meerhaeghe, Alain Le Moine

**Affiliations:** Department of Nephrology, Hôpital Erasme, Hôpital Universitaire de Bruxelles, université libre de Bruxelles, Brussels, Belgium

**Keywords:** acute kidney injury (AKI), immune checkpoint inhibitors, kidney biopsy, hyponatremia, interstitial nephritis

## 1 Introduction

Immune checkpoint inhibitors (ICI) have revolutionized cancer treatment with significant survival benefits. By enhancing the host immune system, ICI can cause immune-related adverse events (irAE). These side effects have common features with auto-immune disease and can virtually affect any organ. The involvement of the kidneys is rare, but it can impact patients' prognosis, treatment, and kidney function. Current knowledge of the mechanisms of toxicity, management, complications, and related biomarkers is scarce. Our Research Topic aimed to explore the recent advances in the field of ICI related nephrotoxicity to improve our knowledge of the disease for better diagnosis, management, treatment, and prevention of ICI induced acute kidney injury (ICI-AKI).

## 2 Editorial of the Research Topic

Catalano et al. published an up-to-date review on the incidence, risk factors, and therapeutic strategies of ICI-AKI. The incidence of ICI-AKI is low but can reach up to 2.2% and is higher with combination therapy. Renal damage caused by anti-CTLA-4 occurs earlier than anti-PD-1/anti-PD-L1 nephrotoxicity on an average of 6–12 weeks compared to 3–12 months. The most common histological lesion seen on kidney biopsy is acute interstitial nephritis (ATIN), but electrolyte abnormalities and glomerular disease may also be seen in a minority of cases. No clinical feature reliably defines ICI-AKI, but prior or concomitant irAE, eosinophilia, sterile pyuria, and sub nephrotic proteinuria may raise suspicion of ICI-AKI due to ATIN. Risk factors for the development of ICI-AKI are underlying chronic kidney disease and the use of medications known to cause ATIN mostly proton-pump inhibitors. Treatment is based on withdrawal of ICI and administration of corticosteroids for 4–6 weeks for severe toxicity (≥grade 2). Second-line immunosuppression may be used for resistant forms. Permanent discontinuation of ICI can have serious implications on patient survival and re-challenge should be considered as only 20–25% of patients relapse ICI-AKI. They conclude that the discovery of non-invasive biomarkers is of utmost importance to diagnose ICI-AKI because of the lack of sensitive or specific clinical features to diagnose ICI-AKI.

In their review, Moss and Perazella describe the clinical and histopathological diagnosis of ICI-AKI. The role of kidney biopsy has been debated and no consensus exists. The authors suggest that a kidney biopsy may help in the definitive diagnosis of ICI–AKI and avoid unnecessary corticosteroid exposure. However, kidney biopsy should not delay corticosteroid treatment if there is a strong suspicion of ICI-AKI and is withheld in patients with contra-indication for kidney biopsy. The authors further suggest the use of the Kidney Disease Improving Global Outcomes (KDIGO) AKI classification rather than the National Cancer Institute's Common Terminology Criteria for Adverse Events (CTCAE). The KDIGO stratifies AKI according to changes in serum creatinine and not changes compared to the upper limit of normal as oncological patients may be cachectic and serum creatinine may not break the upper limit of normal.

Tampe, Kopp et al. describe the compartmentalization of renal PD-L1 expression in injured kidneys in ICI-AKI, its association with PD-1 expression, and clinical findings. They include 7 kidney biopsies of patients with proven ICI-AKI and performed PD-L1 and PD-1 staining. All ICI-AKI showed positive tubular staining for PD-L1, whereas glomerular and endothelial PD-L1 positivity was only seen in 71.4% and 42.9% respectively. Interestingly, the intensity of tubular PD-L1 staining correlated with C-reactive protein (CRP) level. On the other hand, glomerular PD-L1 staining inversely correlated with serum creatinine and low complement C4 levels, and endothelial PD-L1 staining correlated with low complement C4 and LDH. Therefore, the localization of PD-L1 may be influenced by inflammatory markers or complement activation. PD-1 staining was present in interstitial cells and was not associated with PD-L1 staining or localization. However, the intensity of PD-1 staining correlated with the severity of kidney injury. On the transcriptomic level, the expression of PD-1 and PD-L1 are associated with distinct signaling pathways. More research is necessary to unravel the pathogenesis of ICI-AKI, but this study adds additional information on the possible role of PD-1 positive interstitial cells, the severity of kidney injury, and the possible role of the complement system.

Tampe, Baier et al. also contributed a small retrospective study in our research section where they aimed to identify parameters associated with kidney function recovery in their previously established cohort of patients. In total, 6 patients with biopsy-proven ATIN were followed for a mean time of 812 days. They demonstrated that the abundance of intra-renal PD-1/PD-L1, the cumulative steroid dose, and chronic lesions (glomerulosclerosis of IF/TA) on kidney biopsy did not correlate with eGFR change during follow-up. However low sodium levels and high platelet count at the time of biopsy were the strongest predictor of renal function recovery. Higher sodium levels have been associated with the progression of chronic kidney disease and have been shown to enhance transcription induction of pro-fibrotic factors. Salt restriction during AKI recovery may be an attractive therapeutic intervention, but larger and more prospective trials are necessary to confirm these findings.

## Author contributions

TV: Writing – original draft. AL: Writing – review & editing.

